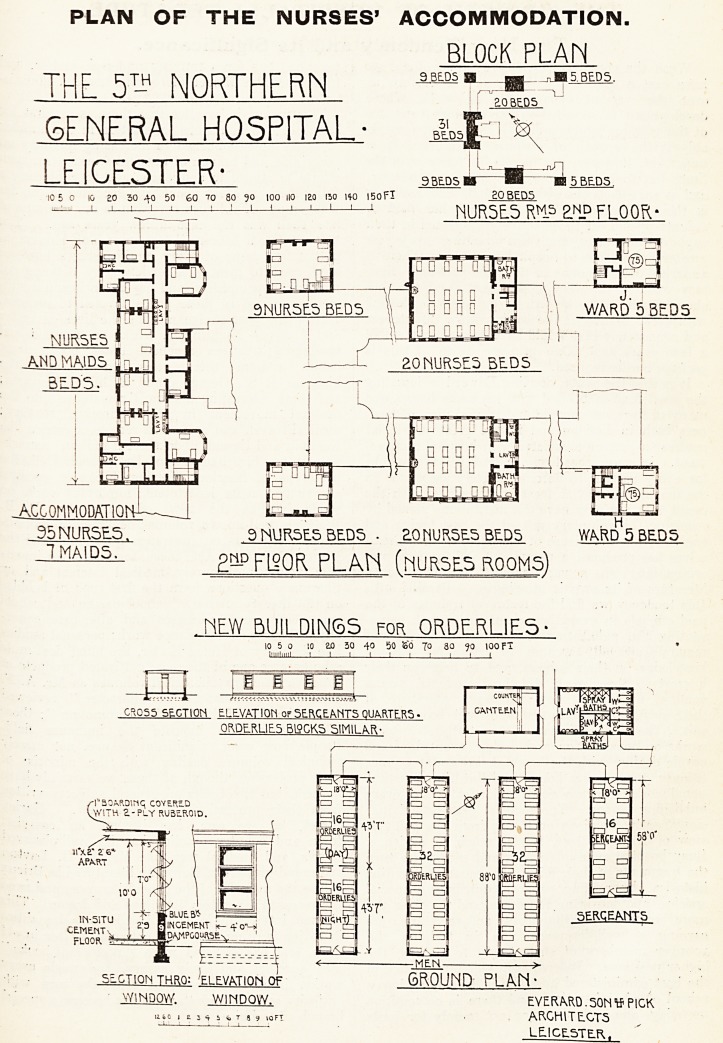# The Territorial Hospital, Leicester

**Published:** 1914-10-10

**Authors:** 


					38 THE HOSPITAL October 10, 1914.
THE TERRITORIAL HOSPITAL, LEICESTER.
THE ACCOM VIODATION FOR NINETY-FIVE NURSES.
The modern hospital has enormously developed
the training of nurses, and the provisions for the'r
accommodation. In such a hospital every nurse is
provided with a separate bedroom. The units which
include the teaching school accommodation, the
model kitchen, the dining-hall, the library, quiet
rooms for study, sitting-rooms for each grade of
nurses, etc., attain so high a standard of hygienic
efficiency and personal comfort as to render a close
study of the accommodation provided for the nurses
at a Territorial hospital as exhibited on the block-
plan on page 39 of surpassing interest. That
plan at once recalls the fact that the Territorial
hospital is a building temporarily devoted to the
treatment of patients, whose qualifications for ad-
mission are supplied by a terrible war. In other
words, every nurse who gives up her comforts in a
civil hospital and joins the staff of a Territorial
hospital must be prepared at once to be brought
face to face with the altered conditions in her
personal surroundings which war entails. We re-
frain from going into details further than to ask
every sister, every nurse and every hospital
committee-man, matron and officer to study this
plan and to compare the immense difference in the
conditions of comfort of a staff where each member
has a separate bedroom and of the inmate who
shares a room some forty feet square with twenty
nurses. The architect is to be commended for the
ingenuity with which he has managed to provide
beds for ninety-five nurses in the scattered buildings
he had to adapt for the purpose. For the rest the
facts and materials we are able to publish make
the exodus of the nurses from the civil hospitals to
the war centres a subject which should give hospital
people everywhere pause to think.
THF 5? NORTHERN GENERAL.
HOSPITAL* LEICESTER-
GROUND FLOOR PLAN *
OcfoBER 10, 1914. THE HOSPITAL 39
PLAN OF THE NURSES' ACCOMMODATION.
BLOCK PLAN
THE 5? NORTHERN
GENERAL HOSPITAL
9 BELD5 m tew ?_rJB5. BEDS.
- !E?I1 s
ao BED5
LEICESTER
5 BEDS.
10 5 O 10 10 30 ^-0 50 60 70 80 ?0 |00 110 IEO 150 HO I50FI 20 BEDS
_L I I I I I I I 1 i I 1 1 1 !
NUR5E.5RM5 ?n_d floor-
5)Q
OS
H
35 NURSES. 3 NURSES BEDS ? 2.0NURSES BEDS WARD 5 BEDS
T MAIDS.
4U-
g^FlSOR FLAN (HUR5ES ROOMS)
HEW BUILDINGS for ORDERLIES
10 5 0 10 2.0 50 40 bo iso Jo 30 70 100 ft
illllllllll I I I I ! I I I I I
1 1 i
0*055 SECTION ELEVATION of SERCEANTS QUARTERS
ORDERLIES B19GK5 SIMILAR-
rrSOAROinq COVERED
.WITH e-PLY RU3E.R01D.
SECTION THRO: 'ELEVATION Of GROUND PLAN'
WINDOW. WINDOW. EVER.ARD.50NIf PICK
1 i u s 4 r j j 10ft ARCHITECTS
^ " ' ' LEICESTER,

				

## Figures and Tables

**Figure f1:**
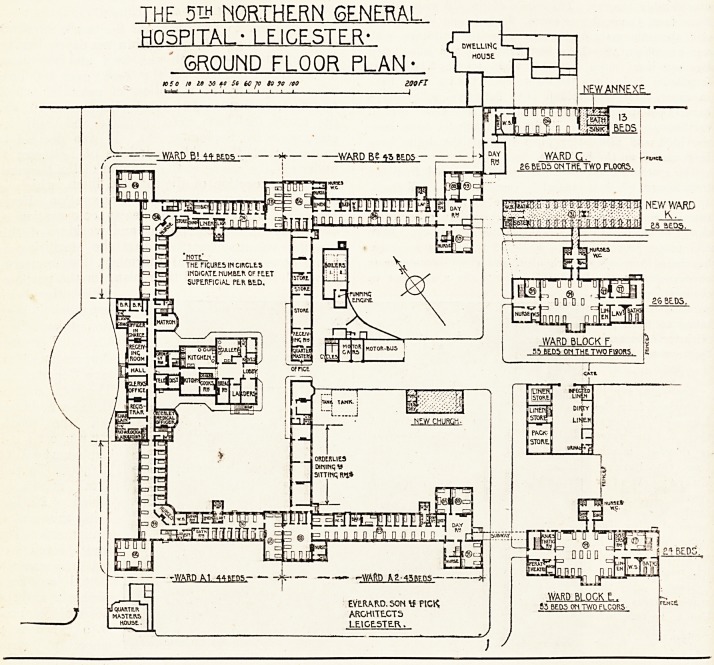


**Figure f2:**